# Participation of Patients From Racial and Ethnic Minority Groups in Phase 1 Early Cancer Drug Development Trials in the US, 2000-2018

**DOI:** 10.1001/jamanetworkopen.2022.39884

**Published:** 2022-11-03

**Authors:** Hayley Dunlop, Evelyn Fitzpatrick, Kevin Kurti, Stephanie Deeb, Erin F. Gillespie, Laura Dover, Divya Yerramilli, Scarlett Lin Gomez, Fumiko Chino, C. Jillian Tsai

**Affiliations:** 1 The Ohio State University College of Medicine, Columbus; 2 Hunter College High School, New York, New York; 3 Icahn School of Medicine at Mount Sinai, New York, New York; 4 Department of Radiation Oncology, Memorial Sloan Kettering Cancer Center, New York, New York; 5 Department of Epidemiology and Biostatistics, University of California, San Francisco; 6 Radiation Medicine Program, Princess Margaret Cancer Centre, Toronto, Ontario, Canada

## Abstract

**Importance:**

Despite federal initiatives encouraging the enrollment of individuals from racial and ethnic minority groups in US clinical trials, no studies to date have specifically examined demographic disparities among participants in phase 1 drug development trials for patients with metastatic cancer.

**Objective:**

To assess trends in the enrollment of patients from racial and ethnic minority groups in US phase 1 therapeutic drug trials for metastatic cancer from 2000 to 2018.

**Design, Setting, and Participants:**

In this cross-sectional study, ClinicalTrials.gov was queried in July 2021 to identify completed phase 1 drug trials for metastatic cancer in the US from January 1, 2000, to December 31, 2018, with published results, yielding 221 phase 1 trials with 8309 participants aged 18 years or older with metastatic solid tumors. Proportions of each racial and ethnic group of trial participants were compared with that from the North American Association of Central Cancer Registries’ Cancer in North America (CiNA) database. Statistical analysis was performed from July 12, 2021, to March 15, 2022.

**Main Outcomes and Measures:**

For each racial and ethnic group, the difference between trial and CiNA proportions was examined using a 2-sample test for equality of proportions with continuity correction.

**Results:**

The 8309 phase 1 trial participants (4198 men [50.5%]; median age, 59 years) included 23 American Indian or Alaska Native participants (0.3%), 371 Asian or Pacific Islander participants (4.5%), 514 Black participants (6.2%), 401 of 5076 Hispanic or Latinx participants (7.9%), and 7154 White participants (86.1%). Industry funded 165 of the 221 trials (74.7%). White patients were overrepresented overall compared with the corresponding CiNA cohort (7154 of 8309 [86.1%] vs 4 113 096 of 4 891 486 [84.1%]; difference, 2.0 percentage points; *P* < .001). There was an increase in overrepresentation of White patients from 2000 to 2011 (trials, 2780 of 3245 [85.7%]; CiNA, 2 378 019 of 2 800 711 [84.9%]; difference, 0.8 percentage points; *P* = .23) to 2012-2018 (trials, 4374 of 5063 [86.4%]; CiNA, 1 735 077 of 2 090 775 [82.9%]; difference, 3.5 percentage points; *P* < .001) and corresponding worsening representation of American Indian or Alaska Native patients (2000-2011: trials, 10 of 3245 [0.3%]; CiNA, 10 905 of 2 800 711 [0.4%]; difference, −0.08 percentage points; 2012-2018: trials, 13 of 5063 [0.3%]; CiNA, 9484 of 2 090 775 [0.5%]; difference, −0.20 percentage points), Asian or Pacific Islander patients (2000-2011: trials, 121 of 3245 [3.7%]; CiNA, 75 033 of 2 800 711 [2.7%]; difference, 1.1 percentage points; 2012-2018: trials, 151 of 5063 [3.0%]; CiNA 70 535 of 2 090 775 [3.4%]; difference, −0.75 percentage points), Black patients (2000-2011: trials, 244 of 3245 [7.5%]; CiNA, 322 701 of 2 800 711 [11.5%]; difference, −4.0 percentage points; 2012-2018: trials, 270 of 5063 [5.3%]; CiNA, 255 625 of 2 090 775 [12.2%]; difference, −6.9 percentage points), and Hispanic or Latinx patients (2000-2011: trials, 161 of 1792 [9.0%]; CiNA, 169 297 of 2 800 711 [6.0%]; difference, 3.0 percentage points; 2012-2018: trials, 240 of 3295 [7.3%]; CiNA, 156 118 of 2 090 775 [7.5%]; difference, −0.2 percentage points). Similar disparities were observed when comparing industry-funded and academic center–sponsored trials.

**Conclusions and Relevance:**

In this cross-sectional study of participants in phase 1 clinical trials of drugs for metastatic cancer, worsening disparities were observed over time in the accrual of patients from racial and ethnic minority groups. These findings may represent widening inequalities in access to trial sites and worsening systemic biases. More efforts are needed to diversify phase 1 cancer drug trials to improve equity in access to new treatments and to ensure that safety and efficacy findings from early drug trials are generalizable across populations.

## Key Points

**Question:**

Does the enrollment of patients from racial and ethnic minority groups in US phase 1 drug trials for metastatic cancer reflect the makeup of the US population with metastatic cancer?

**Findings:**

This cross-sectional study compared the proportions of racial and ethnic groups in 221 phase 1 trials from 2000 to 2018 with population-based cancer registry data and found worsening underrepresentation of American Indian or Alaska Native, Asian or Pacific Islander, Black, and Hispanic or Latinx participants over time.

**Meaning:**

This study suggests that there need to be more efforts to diversify participation in early cancer drug clinical trials to ensure that safety and efficacy findings are generalizable across racial and ethnic populations.

## Teaser

This cross-sectional study assesses trends in the enrollment of patients from racial and ethnic minority groups in US phase 1 therapeutic drug trials for metastatic cancer from 2000 to 2018.

## Introduction

The National Institutes of Health (NIH) and the US Food and Drug Administration have advocated for equitable representation of racial and ethnic groups in drug development trials since the introduction of the Revitalization Act of 1993.^1,2,3^ Since then, numerous studies continued to show a lack of participant diversity in cancer trials, with most of these studies focusing on trials for individual cancer sites or phase 3 trials only.^4,5^

To our knowledge, no studies to date have specifically examined demographic disparities among participants in phase 1 drug development trials. If cancer drugs are not tested in a representative population, the safety or efficacy of these drugs may not be generalizable.^6^ This study sought to assess the distribution of and changes in enrollment of patients from racial and ethnic minority groups in US phase 1 early drug trials for metastatic cancer treatment from 2000 to 2018 because patients with metastatic cancer may benefit most from early drug trials.^7^ We hypothesized that there would be continued overrepresentation of White participants in such trials.

## Methods

In this cross-sectional study, ClinicalTrials.gov was queried in July 2021 using the term: *metastatic cancer*; study type: *interventional studies (clinical trials)*; phase: *early phase 1* and *phase 1* study results *with results*; and country: *United States* published from January 1, 2000, to December 31, 2018. Parameters used to exclude trials included the status “not yet recruiting.” Combined phase 1 and phase 2 trials were included if the phase 1 data were reported separately from phase 2 data. The Strengthening the Reporting of Observational Studies in Epidemiology (STROBE) reporting guideline was used when reporting the results of this cross-sectional study. No institutional review board approval was necessary for this analysis of deidentified, publicly available data per Common Rule 45 CFR 46.102.

Demographic information (race, ethnicity, sex, and age) and trial characteristics (eg, cancer type and inclusion criteria) were identified by reviewing the ClinicalTrials.gov database and publications. The Cancer in North America (CiNA) database was used to estimate the incidence of race and ethnicity in the US adult population (≥18 years) with metastatic cancer between the years 2000 and 2018. The CiNA database represents 97.4% of the US population through combined central state registries, which reflect the demographic diversity of the US population. Race and ethnicity were considered separately.

### Statistical Analysis

Statistical analysis was performed from July 12, 2021, to March 15, 2022. Racial and ethnic groups were categorized in our study according to the classifications in the CiNA database and the ClinicalTrials.gov database. Proportions accounting for the sample size of each trial were calculated for each racial and ethnic group by combining the total number of participants for each racial and ethnic group and dividing by the total number of trial participants. We then compared the proportions of each racial and ethnic group of trial participants with those of corresponding annual incidence data from CiNA; differences with 95% CIs were estimated using a 2-sample test for equality of proportions with continuity correction. This outcome is reported as percentage difference between 2000 to 2011 and 2012 to 2018, with positive percentage differences indicating overrepresentation and negative percentage differences indicating underrepresentation. Ratios of trial participants to incident cancers in the CiNA database by race and ethnicity were calculated, with values greater than 1 indicating overrepresentation and values less than 1 indicating underrepresentation. Pearson χ^2^ tests were used to assess whether the observed number of trial participants of a given race or ethnicity differed significantly from the number estimated from CiNA. Differences were considered to be statistically significant at the *P* < .05 level. All statistical analyses were completed using R Studio, version 1.3.1093 (R Group for Statistical Computing).

## Results

**Figure 1.**
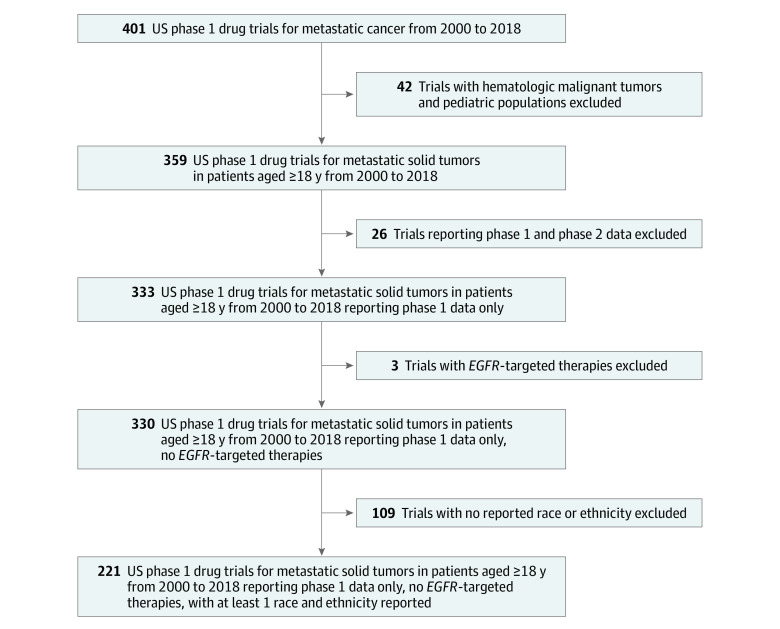
Cohort Selection Flowchart

Of 401 identified trials, 359 focused on metastatic solid tumors in patients aged 18 years or older, and 333 of these reported phase 1 data only (Figure 1). Three trials specifically examining *EGFR* (OMIM 211980)–positive non–small cell lung cancers were excluded because of the disproportionate distribution of *EGFR* variants in Asian female patients.^8^ Of these 330 eligible trials, 221 reported at least 1 race and ethnicity, representing 8309 patients (4198 men [50.5%]; median age, 59 years). No trials identified in the year 2000 reported race or ethnicity. We extracted data from the CiNA database in increments of 2000 to 2011 and 2012 to 2018 to distribute a fairly even number of trials across each time period (2000-2011, 100 trials and 3245 participants; 2012-2018, 121 trials and 5063 participants).

In the cohort of 221 trials from 2000 to 2018, 165 (74.7%) were industry sponsored, 57 (25.8%) focused on metastatic breast cancer, and 34 (15.4%) focused on metastatic colorectal cancer. Of the 8309 total participants, 23 (0.3%) were American Indian or Alaska Native, 371 (4.5%) were Asian or Pacific Islander, 514 (6.2%) were Black, 401 of 5076 (7.9%) were Hispanic or Latinx, and 7154 (86.1%) were White (Table).

**Table.** Demographic Characteristics of Participants in All Phase 1 Trials, Industry-Funded Trials, and Trials Conducted at Academic Centers^a^

Characteristic	Participants, No. (%)				
	American Indian or Alaska Native	Asian or Pacific Islander	Black	Hispanic or Latinx	White
Phase 1 trials					
All (N = 8309)	23 (0.3)	371 (4.5)	514 (6.2)	401 (7.9)	7154 (86.1)
2000-2011 (n = 3245)	10 (0.3)	121 (3.7)	244 (7.5)	161 (9.0)	2780 (85.7)
2012-2018 (n = 5063)	13 (0.3)	151 (3.0)	270 (5.3)	240 (7.3)	4374 (86.4)
Industry trials					
All (n = 7365)	19 (0.3)	332 (4.5)	414 (5.6)	359 (8.3)	6387 (86.7)
2000-2011 (n = 2737)	10 (0.4)	106 (3.9)	178 (6.5)	131 (9.4)	2370 (86.6)
2012-2018 (n = 4628)	9 (0.2)	226 (4.9)	236 (5.1)	228 (7.7)	4017 (86.8)
Academic center trials					
All (n = 5951)	16 (0.3)	221 (3.7)	374 (6.3)	276 (7.8)	5175 (87.0)
2000-2011 (n = 1806)	7 (0.4)	68 (3.8)	149 (8.3)	62 (7.4)	1536 (85.0)
2012-2018 (n = 4145)	9 (0.2)	153 (3.7)	225 (5.4)	214 (8.0)	3639 (87.8)

^a^ Not every trial that reported race reported ethnicity. The number of participants in trials that reported ethnicity was 5076, which is the denominator for all phase 1 trials (all trials in 2000-2011 [n = 1792] and 2012-2018 [n = 3295], all industry-funded trials in 2000-2011 [n = 138] and 2012-2018 [n = 2958], and all trials at academic centers in 2000-2011 [n = 841] and 2012-2018 [n = 2682]).

**Figure 2.**
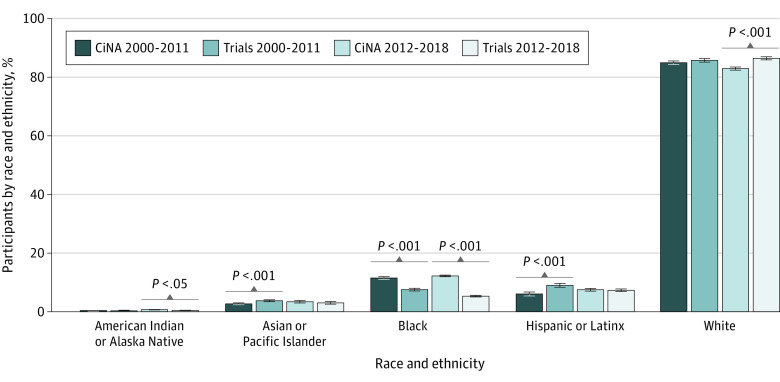
Incidence by Race and Ethnicity in Phase 1 Cancer Drug Trials Compared With Cancer in North America (CiNA) Incident Cancer Cases, 2000-2018

**Figure 3.**
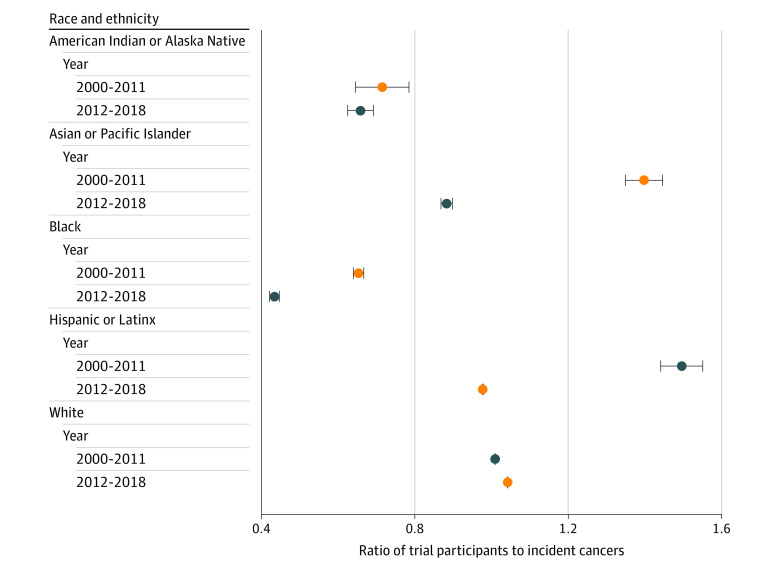
Ratio of Trial Participants to Incident Cancers in the Cancer in North America Database by Race and Ethnicity, 2000-2018 Error bars indicate the 95% CIs for the ratio of the difference in proportions.

Comparing the overall racial and ethnic distributions of trial participants with the corresponding cancer incidence from CiNA, we observed an overrepresentation of White participants (trials, 7154 of 8309 [86.1%]; CiNA, 4 113 096 of 4 891 486 [84.1%]; difference, 2.0 percentage points; *P* < .001) and an underrepresentation of Asian or Pacific Islander participants (trials, 272 of 8308 [4.5%]; CiNA, 145 568 of 4 891 486 [5.9%]; difference, −1.5 percentage points; *P* < .001) and Black participants (trials, 514 of 8308 [6.2%]; CiNA, 578 326 of 4 891 486 [11.8%]; difference, −5.6 percentage points; *P* < .001) during the entire study period. When comparing data from 2000 to 2011 with data from 2012 to 2018, there was an increase in the overrepresentation of White patients from 2000 to 2011 (trials, 2780 of 3245 [85.7%]; CiNA, 2 378 019 of 2 800 711 [84.9%]; difference, 0.8 percentage points; *P* = .23) to 2012 to 2018 (trials, 4374 of 5063 [86.4%]; CiNA, 1 735 077 of 2 090 775 [82.9%]; difference, 3.5 percentage points; *P* < .001) and corresponding worsening representation of American Indian or Alaska Native patients (2000-2011: trials, 10 of 3245 [0.3%]; CiNA, 10 905 of 2 800 711 [0.4%]; difference, −0.08 percentage points; 2012-2018: trials, 13 of 5063 [0.3%]; CiNA, 9484 of 2 090 775 [0.5%]; difference, −0.20 percentage points), Asian or Pacific Islander patients (2000-2011: trials, 121 of 3245 [3.7%]; CiNA, 75 033 of 2 800 711 [2.7%]; difference, 1.1 percentage points; 2012-2018: trials, 151 of 5063 [3.0%]; CiNA 70 535 of 2 090 775 [3.4%]; difference, −0.75 percentage points), Black patients (2000-2011: trials, 244 of 3245 [7.5%]; CiNA, 322 701 of 2 800 711 [11.5%]; difference, −4.0 percentage points; 2012-2018: trials, 270 of 5063 [5.3%]; CiNA, 255 625 of 2 090 775 [12.2%]; difference, −6.9 percentage points), and Hispanic or Latinx patients (2000-2011: trials, 161 of 1792 [9.0%]; CiNA, 169 297 of 2 800 711 [6.0%]; difference, 3.0 percentage points; 2012-2018: trials, 240 of 3295 [7.3%]; CiNA, 156 118 of 2 090 775 [7.5%]; difference, −0.2 percentage points) (Figure 2). Similar worsening of disparities was observed for both industry-funded and academic center–sponsored trials (Table). Increasing disparities over time by race and ethnicity for all trials, especially Asian and Pacific Islander patients (ratio of trial to CiNA participants, 1.44 in 2000-2011 to 0.87 in 2012-2018), Black patients (0.64 in 2000-2011 to 0.42 in 2012-2018), and Hispanic or Latinx patients (1.55 in 2000-2011 to 0.98 in 2012-2018), are shown in Figure 3. Results for sensitivity analyses for trials targeting *EGFR*-positive cancers are included in the eTable and eFigure in the Supplement.

## Discussion

Although the benefits of phase 3 clinical trials to patients are widely accepted, there has been debate regarding the expected benefits associated with phase 1 trial participation.^7^ Although personal therapeutic gain should not be expected from enrollment in phase 1 trials, some benefits of quality of life and access to palliative care have been documented.^7^ To improve equity in drug design and testing, phase 1 cancer trials should be accessible to any patient who is eligible, regardless of their demographic background.

This study highlights disparities in the enrollment of patients from racial and ethnic minority groups in phase 1 drug trials for metastatic cancer; this gap appears to be worsening over time. Inclusion of patients from racial and ethnic minority groups in phase 1 cancer trials was worse than observed in phase 3 trials, especially among Black participants (−6.9% difference in Black participants in phase 1 trials in 2012-2018 compared with −2.6% difference in phase 3 trials since year 2011).^4,9^ Similar disparities in phase 1 cancer clinical trial enrollment have been noted in studies conducted at single institutions.^10^

When examining racial and ethnic disparities in clinical research, it is essential to acknowledge the harms that individuals of racial and ethnic minority groups have experienced at the hands of the US health care system, especially in the research setting.^11^ These historical harms have led to mistrust of academic and research institutions and investigators, which is further exacerbated by current socioeconomic and health care system inequities.^11^ These findings may represent systemic biases, a lack of early drug development initiatives that are more inclusive, mistrust of clinical researchers among racial and ethnic minority groups, and a lack of access and outreach of clinical trial accrual sites.^4,5,7,11^

Given that structural factors may lead to variable distribution of comorbidities across demographic groups, researchers should consider their inclusion and exclusion criteria carefully and avoid arbitrary or historic standards because these standards may negatively impact trial diversity.^12^ Collecting data on social determinants of health for trial participants may provide a better understanding of the barriers to trial participation for patients who belong to minority racial and ethnic groups and will help researchers address these barriers in future studies.^12^ Interventions such as providing transportation, meal vouchers, and childcare support have been suggested as ways in which to increase patient accrual and engagement if these social determinants of health impact potential trial participants.^12^

Phase 1 cancer trials are often conducted at academic institutions because the logistics of running early drug trials make them difficult to conduct in community settings. Because patients from racial and ethnic minority groups may be less likely to receive care at academic medical centers,^13^ this could explain accrual disparities. Clinical innovations, such as hospital-at-home initiatives and establishing community-academic alliances, may lead to higher trial enrollment in community-based partner sites and expand access to phase 1 trial drugs to patients with cancer who may not regularly obtain care at academic medical centers.^14,15^ Using telemedicine platforms to offer virtual visits when available, establishing direct-to-patient shipping for oral therapeutics, and forming partnerships between advocacy groups, trial sponsors, and research teams to expedite accrual to studies may also help lessen disparities.^16^

Finally, improved reporting of sociodemographic data is needed for future clinical trials. The NIH Policy on the Dissemination of NIH-Funded Clinical Trial Information, which went into effect on January 18, 2017, requires investigators and sponsors to report the race and ethnicity of participants to ClinicalTrials.gov if that information is collected.^17^ Although this policy has improved the reporting of race and ethnicity for clinical trials, more work is needed to standardize the collection of sociodemographic data for clinical trials so that equity and inclusion can be assessed in future studies.^17^

### Limitations

This study has some limitations. The greatest limitation is the number of trials not reporting race and ethnicity (32.2%). Because all trial data were reported at the aggregate level, we were unable to examine the association of the intersectionality of sex, age, and socioeconomic status with racial and ethnic disparities in trial accrual. CiNA data present incident and unique cases, but patients may have participated in multiple clinical trials and therefore may be captured multiple times within ClinicalTrials.gov. The age distribution of trial participants also may differ from CiNA incident cases.

## Conclusions

In this cross-sectional study of participants in phase 1 clinical trials of drugs for metastatic cancer, persistent and worsening disparities in the accrual of patients from racial and ethnic minority groups were observed. These include underenrollment of Asian or Pacific Islander patients, Black patients, and Hispanic or Latinx patients in clinical trials overall and underenrollment of American Indian or Alaska Native patients, Asian or Pacific Islander patients, Black patients, and Hispanic or Latinx patients in trials conducted at academic institutions. These findings may represent widening inequalities in access to trial sites and worsening systemic biases. Current efforts to diversify clinical trials should expand their reach to include phase 1 cancer drug trials to improve equity in access to new treatments at every stage and to ensure that safety and efficacy findings from early cancer drug trials are generalizable across populations.

## Notes

Supplement. **eTable.** Demographics of Participants in All Phase I Trials, Industry Funded Trials, and Trials Conducted at Academic Centers Including Trials Targeting EGFR+ Non-Small Cell Lung Cancers **eFigure.** Incidence by Race and Ethnicity in All Phase I Trials Including Trials Targeting EGFR+ Non-Small Cell Lung Cancers
