# Supplementary material for: Participation of Patients From Racial and Ethnic Minority Groups in Phase 1 Early Cancer Drug Development Trials in the US, 2000-2018
Source: JAMA Netw Open. 2022 Nov 3;5(11):e2239884. doi: 10.1001/jamanetworkopen.2022.39884 (PMC9634497; doi:10.1001/jamanetworkopen.2022.39884)
Supplement: Supplement. — eTable. Demographics of Participants in All Phase I Trials, Industry Funded Trials, and Trials Conducted at Academic Centers Including Trials Targeting EGFR+ Non-Small Cell Lung Cancers eFigure. Incidence by Race and Ethnicity in All Phase I Trials Including Trials Targeting EGFR+ Non-Small Cell Lung Cancers [file jamanetwopen-e2239884-s001.pdf]

Supplementary Online Content

Dunlop H, Fitzpatrick E, Kurti K, et al. Participation of patients from racial and ethnic minority groups in phase 1 early cancer drug development trials in the US, 2000-2018. *JAMA Netw Open.* 2022;5(11):e2239884. doi:10.1001/jamanetworkopen.2022.39884

eTable. Demographics of Participants in All Phase I Trials, Industry Funded Trials, and Trials Conducted at Academic Centers Including Trials Targeting EGFR+ Non-Small Cell Lung Cancers
eFigure. Incidence by Race and Ethnicity in All Phase I Trials Including Trials Targeting EGFR+ Non-Small Cell Lung Cancers

This supplementary material has been provided by the authors to give readers additional information about their work.

© 2022 Dunlop H et al. *JAMA Network Open.*

eTable. Demographics of Participants in All Phase I Trials, Industry Funded Trials, and Trials Conducted at Academic Centers Including Trials Targeting EGFR+ Non-Small Cell Lung Cancers

	AI/AN	API	Black	H/L	White
**All Phase I** (n=9,273)	24 (0.26%)	870 (9.4%)	531 (5.7%)	405 (4.4%)	7560 (81.5%)
**2001-2011** (n=3,387)	10 (0.3%)	126 (3.7%)	253 (7.5%)	164 (8.9%)	2906 (85.8%)
**2012-2018** (n=5,880)	14 (0.24%)	744 (12.7%)	278 (4.7%)	241 (7.3%)	4654 (79.1%)
**Industry** (n=8,215)	20 (0.24%)	824 (10.0%)	425 (5.2%)	359 (8.2%)	6698 (81.5%)
**2001-2011** (n=2,971)	10 (0.33%)	107 (3.6%)	181 (6.1%)	131 (9.4%)	2418 (86.6%)
**2012-2018** (n=5,426)	10 (0.18%)	717 (13.2%)	244 (4.5%)	288 (7.7%)	4280 (78.9%)
**Academic** (n=7,724)	17 (0.22%)	822 (10.6%)	436 (5.6%)	392 (9.1%)	6426 (83.2%)
**2001-2011** (n=2,826)	10 (0.35%)	111 (3.9%)	205 (7.3%)	139 (9.0%)	2425 (85.8%)
**2012-2018** (n=5,122)	7 (0.14%)	711 (13.9%)	231 (4.5%)	163 (6.1%)	4001 (78.0%)

© 2022 Dunlop H et al. *JAMA Network Open.*

eFigure. Incidence by Race and Ethnicity in All Phase I Trials Including Trials Targeting EGFR+ Non-Small Cell Lung Cancers

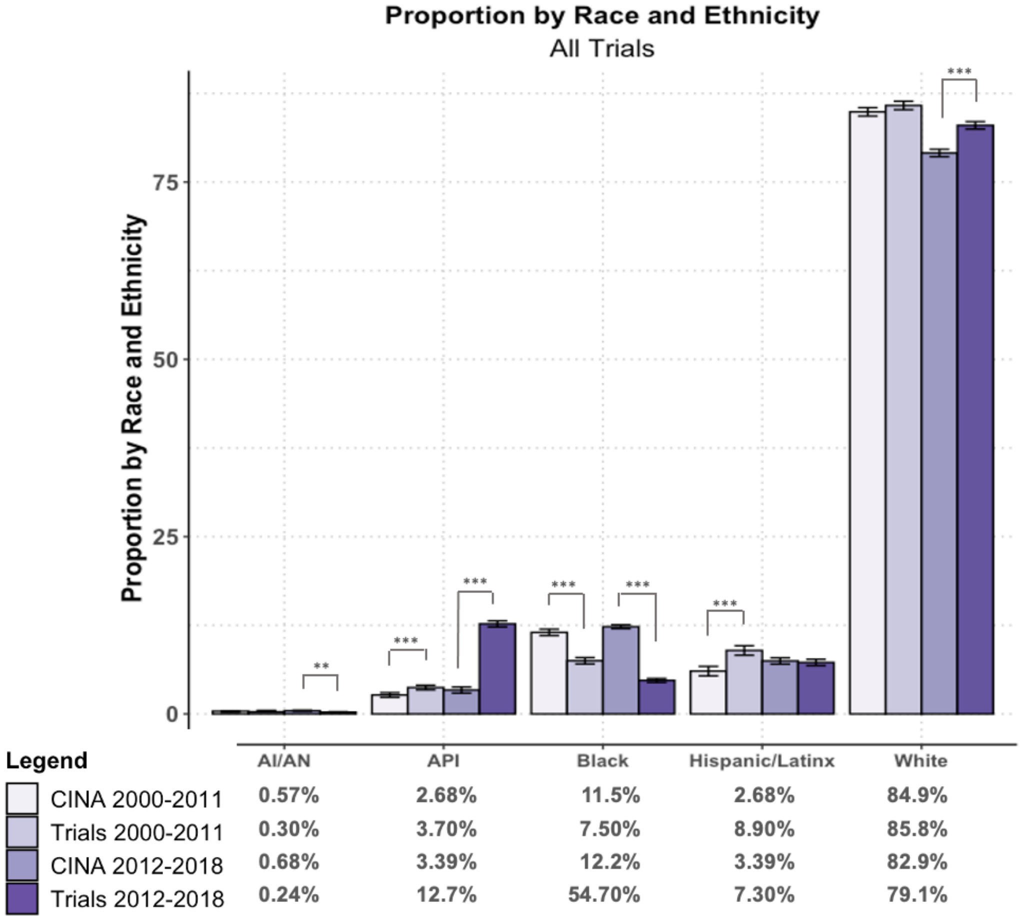

p<0.05*,p<0.01*,p<0.001***

© 2022 Dunlop H et al. *JAMA Network Open.*
